# Supplementary material for: Predicting entrepreneur fundraising success from focus group EEG data
Source: PLoS One. 2026 Jan 27;21(1):e0340606. doi: 10.1371/journal.pone.0340606 (PMC12843520; doi:10.1371/journal.pone.0340606)
Supplement: Attachment — Submitted filename: Response to Reviewers_111525.docx [file pone.0340606.s001.docx]

**Response to Reviewer Comments: Overview**

**We thank the review team for the insightful and encouraging responses to the initial manuscript. We have prepared a revision that addresses the concerns raised in the initial round.**

**Before providing point-by-point responses, we would like to address two prominent and recurring concerns:**

**1. *Reporting power analyses & robustness checks.* Both reviewers felt the work would be strengthened if we could show that our findings are based on power analyses along with some robustness checks, including FAA calculations and/or correcting some multiple comparisons for rolling window analysis. R2 particularly suggested reporting relevant power analyses on sample sizes and some robustness checks.**

**To address these,** we added specific guidelines on the reported sample size (N = 28) and the number of pitch videos (N = 14) used, along with a statistical sensitivity analysis (*p*. 8). We also state explicitly that our findings are exploratory and hypothesis-generating, not confirmatory. We further incorporated robustness checks on FAA calculations by defining different ranges of alpha oscillations (8–12 Hz) and reported bootstrapping analyses on rolling-window correlations between FAA/ISC and population-level investor interest as well as deal outcomes.

**2. *Toning down interpretations and fixed conclusions.* Both reviewers again suggested toning down some strong interpretations from reverse inferencing and/or fixed conclusions.**

**To address this,** we removed interpretations related to psychological mechanisms or reverse inference (FAA as approach motivation; ISC as shared engagement) and instead focused on the predictive roles of these metrics. As suggested by R1, not only we revised the wording in several sections, including the title and results, we overhauled our abstract, introductions and discussions to condense our predictions.

**We hope these clarifications come through in the revised manuscript. Below we provide detailed responses to the concerns and suggestions raised by each reviewer.**

**Response to Reviewer #1**

***Comment:*** *The paper is well-organized and well-written. Exploratory studies such as the current one can be valuable additions to the literature, despite small samples sizes (low power), relatively large p-values, and lack of correction for multiple comparisons, even though they often prove irreplicable (Ioannidis 2005, “Why Most Published Research Findings Are False“, PLOS Medicine). However, fixed conclusions when interpreting such preliminary results would be premature. Major issues:1. Please use past tense when describing results, to avoid the implication that the results are globally applicable to all future studies that test the same hypotheses. Statements in need of reformulation include. Title: Could be more accurately stated as, for example, “Prediction of entrepreneur fundraising success from focus group EEG data,” although the difference is a subtle one.*

**Response:** We thank the reviewer for these helpful suggestions. In the revised manuscript, we have carefully revised all results-related statements to use the past tense, in order to avoid overgeneralization and the implication that findings are universally applicable beyond this study. We also updated the title to “Predicting Entrepreneur Fundraising Success from Focus Group EEG Data” to reflect more accurately the study’s scope and exploratory nature.

More important, we added a statistical sensitivity analysis to clarify the limits of our sample size (N = 28) and number of pitch videos used (N = 14). We now report Monte Carlo simulations (5,000 shuffles) of inter-subject correlation–behavior correlations, showing power estimates for small (ρ = 0.10), medium (ρ = 0.30), and large (ρ = 0.50) effects. We explicitly note that the design is exploratory and hypothesis-generating, rather than confirmatory (*p*. 8).

Regarding multiple-comparison concerns, we now report nonparametric bootstrapping analyses (5,000 iterations) on the rolling-window correlations between FAA/ISC and investor interest or deal outcomes. While these additional analyses validate the temporal robustness of the observed effects accounting for the repeated-window bins, we acknowledge explicitly that nonparametric bootstrapping is not a formal correction method such as False Discovery Rate (FDR). However, given our exploratory design and limited number of stimuli (N = 14), bootstrapping can provide a pragmatic approach to assess the stability and robustness of temporal effects across rolling windows.

***Comment:*** *Results: Sentences/phrases in need of modification include “Thus, greater FAA signals captured in early impressions to persuasive pitches can predict investment interest,” “… is the stronger predictor of aggregate fund amounts,” “These findings suggest that overall FAA signals can predict aggregate fund amounts raised,” and “showing that synchronized neural activity—particularly in the frontal-central regions, averaged across the entire pitch video—is predictive of both deal outcomes and funding amounts.” Discussion: Sentences/phrases in need of modification include “As anticipated, our findings indicate that neural data from a laboratory sample predicts aggregate outcomes,” and “Entrepreneurs can focus on their initial impressions and the delivery of their product solutions to enhance neural synchrony among funders, which correlates with increased investor interest and ultimately securing a deal. In addition, entrepreneurs and trainers can use our neural insights to refine their presentation strategies and improve their chances of closing deals.”*

**Response:** In the revised manuscript, we have carefully revised all results-related statements to use the past tense (e.g., “predicted” instead of “can predict”) to clearly indicate that findings are specific to this study.

We also softened the language in both the *Results* and *Discussion* sections to emphasize the exploratory and predictive—rather than confirmatory—nature of our analyses. For example, we revised phrases such as on pp. 25-26: “Entrepreneurs who emphasize strong initial impressions and the delivery of their product solutions may foster greater neural similarity among potential funders, which, in our study, was associated with increased investor interest and, in some cases, securing a deal. In addition, entrepreneurs and trainers could draw on these neural insights to refine presentation strategies and improve the likelihood of closing deals.”

***Comment:*** *Please be more specific in describing important details of the study in the title and abstract. For example, the “brain signals” discussed are EEG data. The “small laboratory sample” and “out-of-sample investment interest” could also benefit from additional details.*

**Response:** In the revised abstract, we now specify that “brain signals” explicitly refer to electroencephalographic (EEG) data collected from a small laboratory sample (N = 28) who watched 14 Shark Tank pitch videos. These signals were then compared against population-level (or out-of-sample) investor interest ratings (N = 497) and real-world deal outcomes. As previously mentioned, we also revised the title to reflect these details more clearly and accurately.

***Comment:*** *Minor items*:
*1. p4. VC is defined as venture capital, but VCs does not appear to be its plural form.
2. p11. Grammar: “data was.”
3. p14. Why the sudden switch to present tense in “We then derive the PSD reflecting the power at the alpha band frequency and apply a 5-second moving window across the EEG data”?
4. p15. The heading “Controls” might better be described as “Control variables” because the term controls usually refers to participants in a control group.
5. p20. Grammar: “However, when we employed our TFR rolling window technique and none of the time windows of moment-to-moment FAA activity forecasted binary deal outcomes.”
6. p25. Grammar: “While we represent an initial step in neuroforecasting within entrepreneurship, and several questions arise regarding our paradigm for future research.”
7. p25. “experienced vs. angel investors” seems to imply that these are mutually exclusive categories.*

**Response:** We appreciate the reviewer’s close reading and attention to detail. All seven items have been carefully addressed in the revised manuscript. We hope you will be pleased with the revision.

**Response to Reviewer #2**

***Comment:*** *1. Sample size and generalizability. • The lab EEG sample (N=28) is small for inter-subject correlation and synchrony analyses. While the out-of-sample survey (N=497) helps, the EEG-derived predictions might be unstable or overly sensitive to outliers. • Suggestion: report power analysis or bootstrapping stability checks; highlight limits in generalization to broader investor populations (e.g., experienced VCs vs. MTurk participants).*

**Response:** We appreciate the reviewer’s concern regarding sample size and generalizability. The EEG sample (N = 28) indeed warrants careful sensitivity checks. As noted in the response to Reviewer 1 above, we added in the revision a Monte Carlo–based statistical sensitivity analysis (5,000 shuffles) for ISC–behavior correlations, based on 378 pairs (28 x 27 / 2) and 14 pitch videos used in the task. This analysis provides estimated power for small (ρ = 0.10), medium (ρ = 0.30), and large (ρ = 0.50) effects and reinforces the exploratory nature of our design (*p*. 8). Further, we note that our target sample size (N = 28) was guided by prior EEG inter-subject correlation work, a study using 30 participants to predict the success of 24 Spotify music tracks (Leeuwis et al. 2021) (*p*. 8).

Importantly, recent methodological work in advertising research provides an empirical benchmark indicating that our sample size aligns with recommendations for ISC-based analyses. Van Diepen et al. (2024, *Journal of Advertising*) report that ISC is the most reliable EEG metric for dynamic naturalistic videos, achieving acceptable reliability (r > .70) with 90% confidence using approximately 25 participants.

Regarding generalizability, we rephrased our discussion to limit claims to our lab-based focus group and MTurk sample. We discuss ecological constraints such as the use of recorded pitch videos rather than live interactions (see below for more) and recommended future studies involving professional investors and dyadic or live pitch settings to enhance external validity (*p*. 26). However, given the growing trend of entrepreneurs presenting pitch videos on crowdfunding platforms such as Kickstarter, our setup may still hold practical relevance (e.g., grasp early impression, elaborate well on their product or service descriptions), despite its inherent limitations.

***Comment:*** *2. Ecological validity. • Pitches are Shark Tank videos, not live, interactive pitches. EEG signals may differ from actual investor-founder interactions that include Q&A and dynamic feedback. • Suggestion: acknowledge stronger ecological limitations and recommend testing in dyadic or live pitch settings.*

**Response:** We acknowledge the reviewer’s concern regarding ecological validity. In the revised manuscript, we now explicitly state that EEG data were collected while participants viewed recorded Shark Tank pitches, rather than engaging in live, interactive exchanges. This distinction constrains generalizability to real-world investor–founder interactions that typically involve Q&A and/or dynamic feedback (*p*. 26). To address this limitation, we now recommend future studies (1) test dyadic or live pitch settings with structured Q&A, (2) use hyperscanning techniques to capture inter-brain synchrony during interaction/negotiation, or (3) recruit stratified samples, including professional investors, to examine experience effects. These additions are intended to temper our claims and clarify the context-bound nature of the findings (*p*. 26).

***Comment:*** *3. EEG preprocessing and robustness. • Preprocessing is described, but critical details (e.g., ICA rejection thresholds, % variance removed, number of components rejected per subject) are not fully reported. • FAA computation relies on F3/F4; however, alpha asymmetry is notoriously noisy and can be confounded by artifacts (eye movement, muscle tension). • Suggestion: add robustness checks (e.g., alternative reference schemes, different band definitions, inclusion/exclusion of noisy subjects).*

**Response:** We appreciate the reviewer’s attention to EEG processing and robustness. We have expanded the description of preprocessing procedures. Specifically, we now report that EEG data were decomposed into 15 independent components per video epoch. Eye-related components were identified via correlations with 4 EOG channels, using a z-threshold of 1.96, and on average, between 1 and 4 ICA components were excluded per participant and video epoch (*p*. 12). We also uploaded all relevant scripts used in preprocessing, FAA, and neural similarity analyses on OSF.

To address robustness, we ran the two additional checks: (1) we tested slightly varied alpha-band definitions (8–12 Hz vs. 8–13 Hz), which yielded highly similar FAA results, and (2) we applied nonparametric bootstrapping (5,000 iterations) to rolling-window FAA/ISC correlations, reporting stable confidence intervals (CIs) for the early FAA (4–5 secs) and product-solution ISC (~47–49 secs) effects. Together, these additions clarify preprocessing details and confirm that the reported findings are robust across analytic specifications.

***Comment:*** *4. Multiple comparisons / statistical control. • Rolling-window FAA analyses involve many time bins, but correction for multiple comparisons is not clearly described (e.g., cluster-based permutation, FDR). • Synchrony effects are reported around 47–50 seconds — was this result corrected for the fact that many windows were tested? • Suggestion: clarify family-wise error control to strengthen claims.*

**Response:** We thank the reviewer’s point about family-wise error control. We tested multiple-comparison correction methods, including FDR. However, the temporal peaks were not statistically significant after FDR adjustment—likely due to the conservative nature of such corrections given our limited number of stimuli (14 videos). We instead opted to report nonparametric bootstrapping (5,000 iterations) for the rolling-window FAA and ISC analyses, as noted above, which we believe is a more suitable robustness approach for exploratory studies with a limited number of stimuli. Specifically, the bootstrapping procedure resampled correlation distributions across 5,000 iterations to estimate 95% CIs for each temporal window. The results showed that FAA correlations during the 4–5 s window and ISC correlations during the 47–49 s window remained significantly positive, suggesting temporal robustness (*pp*. 19, 21).

***Comment:*** *5. Interpretation risk (“reverse inference”). • FAA is interpreted as “approach motivation” and synchrony as “collective engagement.” These are plausible, but EEG signals are indirect markers and may reflect multiple overlapping processes (e.g., visual attention, motor planning). • Suggestion: soften causal language; frame findings as predictive markers rather than mechanistic explanations.*

**Response:** We recognize the reviewer’s concern regarding interpretation risk and reverse inference. In the revised manuscript, we have removed all causal or mechanistic language referring to FAA as “approach motivation” and ISC as “collective engagement.” These terms now appear only in reference to prior theoretical frameworks, and are not presented as inferred psychological mechanisms based on our own findings. In the discussion section, we explicitly clarify that both FAA and ISC are treated as predictive neural markers, rather than direct indicators of motivational or engagement processes. We now accentuate that ISC can reflect effective communication, as it gradually builds over the course of a compelling pitch.

***Comment:*** *6. Behavioral benchmarks. • Sentiment analysis was limited (VADER, NRCLex) and may not capture nuance (e.g., sarcasm, authenticity). The null findings could reflect weak measures rather than EEG superiority. • Suggestion: consider adding linguistic richness measures (e.g., LIWC categories, narrative complexity) or justify why the chosen sentiment models are adequate.*

**Response:** We appreciate the reviewer’s thoughtful feedback on behavioral benchmarks. In the revised manuscript, we now explicitly justify our use of the Vader and NRCLex sentiment analysis tools, explaining that these lexicon-based approaches are well-suited for analyzing 1–2 minute monologic Shark Tank pitch transcripts, which are designed to convey affective tone rather than complex narrative structure (*pp*. 14-15). We emphasize that our selected measures capture key affective dimensions (e.g., joy, trust, fear) that align with how founders emotionally frame their claims (e.g., excitement, credibility, or risk framing) in short persuasive contexts. While we acknowledge that these tools may not fully capture nuanced linguistic features like sarcasm or authenticity, they offer a practical and interpretable benchmark for affective content in brief pitch formats.

***Comment:*** *Overall, the study is technically sound and provides novel insights into the neural correlates of entrepreneurial success. However, the statistical analyses need more transparency and caution. The main conclusions are supported, but they should be framed more conservatively, especially regarding real-world outcome prediction and the robustness of temporal effects.*

**Response:** We appreciate the reviewer’s encouraging assessment and constructive guidance. We not only overhauled the introduction and discussion parts, but we have enhanced statistical transparency and tempered our interpretations throughout the revised manuscript. Specifically, we (1) expanded reporting of EEG preprocessing steps, power simulations, and bootstrapping procedures to clarify analytic rigor; (2) reframed the findings as exploratory and predictive, rather than confirmatory; and (3) softened language around real-world forecasting, ensuring that claims about outcome prediction are conservatively stated and context-bound. We hope you will be pleased with these revisions.
